# Habitat escalated adaptive therapy (HEAT): a phase 2 trial utilizing radiomic habitat-directed and genomic-adjusted radiation dose (GARD) optimization for high-grade soft tissue sarcoma

**DOI:** 10.1186/s12885-024-12151-7

**Published:** 2024-04-09

**Authors:** Arash O. Naghavi, J. M. Bryant, Youngchul Kim, Joseph Weygand, Gage Redler, Austin J. Sim, Justin Miller, Kaitlyn Coucoules, Lauren Taylor Michael, Warren E. Gloria, George Yang, Stephen A. Rosenberg, Kamran Ahmed, Marilyn M. Bui, Evita B. Henderson-Jackson, Andrew Lee, Caitlin D. Lee, Ricardo J. Gonzalez, Vladimir Feygelman, Steven A. Eschrich, Jacob G. Scott, Javier Torres-Roca, Kujtim Latifi, Nainesh Parikh, James Costello

**Affiliations:** 1 Department of Radiation Oncology, H. Lee Moffitt Cancer Center and Research Institute, Tampa, FL USA; 2 Department of Bioinformatics and Biostatistics, H. Lee Moffitt Cancer Center and Research Institute, Tampa, FL USA; 3 Department of Radiation Oncology and Applied Sciences, Geisel School of Medicine, Dartmouth College, Hanover, NH USA; 4 Department of Radiation Oncology, James Cancer Hospital, The Ohio State University Comprehensive Cancer Center, Columbus, OH USA; 5 Clinical Trials Office, H. Lee Moffitt Cancer Center and Research Institute, Tampa, FL USA; 6 Department of Pathology, H. Lee Moffitt Cancer Center and Research Institute, Tampa, FL USA; 7 Department of Sarcoma, H. Lee Moffitt Cancer Center and Research Institute, Tampa, FL USA; 8 Translational Hematology and Oncology Research, Radiation Oncology Department, Cleveland Clinic, Cleveland, OH USA; 9 Department of Diagnostic Imaging and Interventional Radiology, H. Lee Moffitt Cancer Center and Research Institute, Tampa, FL USA

**Keywords:** Sarcoma, Soft tissue sarcomas, Radiomic, Pathology, Genomic, Neoadjuvant, Radiotherapy, Adaptive therapy, Clinical trial

## Abstract

**Background:**

Soft tissue sarcomas (STS), have significant inter- and intra-tumoral heterogeneity, with poor response to standard neoadjuvant radiotherapy (RT). Achieving a favorable pathologic response (FPR ≥ 95%) from RT is associated with improved patient outcome. Genomic adjusted radiation dose (GARD), a radiation-specific metric that quantifies the expected RT treatment effect as a function of tumor dose and genomics, proposed that STS is significantly underdosed. STS have significant radiomic heterogeneity, where radiomic habitats can delineate regions of intra-tumoral hypoxia and radioresistance. We designed a novel clinical trial, Habitat Escalated Adaptive Therapy (HEAT), utilizing radiomic habitats to identify areas of radioresistance within the tumor and targeting them with GARD-optimized doses, to improve FPR in high-grade STS.

**Methods:**

Phase 2 non-randomized single-arm clinical trial includes non-metastatic, resectable high-grade STS patients. Pre-treatment multiparametric MRIs (mpMRI) delineate three distinct intra-tumoral habitats based on apparent diffusion coefficient (ADC) and dynamic contrast enhanced (DCE) sequences. GARD estimates that simultaneous integrated boost (SIB) doses of 70 and 60 Gy in 25 fractions to the highest and intermediate radioresistant habitats, while the remaining volume receives standard 50 Gy, would lead to a > 3 fold FPR increase to 24%. Pre-treatment CT guided biopsies of each habitat along with clip placement will be performed for pathologic evaluation, future genomic studies, and response assessment. An mpMRI taken between weeks two and three of treatment will be used for biological plan adaptation to account for tumor response, in addition to an mpMRI after the completion of radiotherapy in addition to pathologic response, toxicity, radiomic response, disease control, and survival will be evaluated as secondary endpoints. Furthermore, liquid biopsy will be performed with mpMRI for future ancillary studies.

**Discussion:**

This is the first clinical trial to test a novel genomic-based RT dose optimization (GARD) and to utilize radiomic habitats to identify and target radioresistance regions, as a strategy to improve the outcome of RT-treated STS patients. Its success could usher in a new phase in radiation oncology, integrating genomic and radiomic insights into clinical practice and trial designs, and may reveal new radiomic and genomic biomarkers, refining personalized treatment strategies for STS.

**Trial registration:**

NCT05301283.

**Trial status:**

The trial started recruitment on March 17, 2022.

## Background

Minimal progress has been made in the management of soft tissue sarcoma (STS) over the past several years [1]. The underlying difficulty behind this effort is multifactorial, as sarcoma is a rare disease entity representing approximately 1% of cancer incidence in the United States and possesses significant intra- and inter-tumoral heterogeneity, consisting of dozens of histologies with varying clinical behaviors [2]. Despite this known diversity, the current standard of care is a uniform, conventionally fractionated radiation dose delivered homogenously, followed by a planned operation, regardless of histology, grade, or obvious radiographic intratumoral heterogeneity [3–5]. This treatment algorithm results in local recurrence rates ranging from 10 to 20%, the majority of which occur within the radiotherapy treatment field. In addition, standard neoadjuvant radiation treatment achieves a favorable pathological response (FPR ≥ 95% cellular response) in only 8% of cases [6]. These findings clinically support the claim that STS may be inherently radioresistant [1, 7–9], which has also been described radio-biologically, with an alpha/beta ratio ranging from 2 to 6 [10, 11].

In recent studies, we have developed the genomic adjusted radiation dose (GARD), an RT-specific metric that estimates the clinical effect of a given RT dose for an individual tumor. GARD is based on the gene expression-based radiosensitivity index (RSI), a clinically validated genomic signature of cellular radiosensitivity and the linear quadratic model [12, 13]. In a pan-cancer analysis including 1615 patients in seven different disease sites, GARD outperformed RT dose (EQD2) by its association with clinical outcome (overall survival and recurrence risk), and its prediction and quantification of RT benefit for each individual patient [12]. STS was found to have a broad range of radiosensitivity across various histologies, where the ideal neoadjuvant dose for highly radioresistant STS histologies is a BED_3.29_ ≥97 Gy, which translates to 57.5 Gy in 25 fractions [11]. This is consistent with the improved local control seen with neoadjuvant simultaneous integrated boost in retroperitoneal sarcoma, where 57.5 Gy in 25 fractions had a higher 5-year abdominopelvic control (96% vs. 70%, *p* = 0.046), when compared to standard fractionation RT [14].

Given its ability to quantify RT benefit, we have proposed that GARD could be utilized to design clinical trials. To support this, we have demonstrated that GARD-based model predictions for uniform dose escalation (as in RTOG 0617 for NSCLC) or uniform de-escalation in HPV + oropharynx cancer (as in HN005) align with the clinical results reported for the actual trial. There are two approaches to GARD-based clinical design: (1) Uniform GARD-optimized dose and (2) Personalized GARD-optimized dose. The first approach is focused on diseases where GARD estimates patients are either primarily under/over-dosed with RT and thus a single escalated/de-escalated RT dose is predicted to impact the overall treatment benefit for the population. In the second approach, no single RT dose is identified that can improve the overall outcome of the population and only a patient-specific personalized approach is predicted to work. GARD-optimization models propose that in STS a uniform approach would be successful.

Although, STSs are primarily considered radioresistant, gene expression and RSI variability exists both between tumors and within each tumor [15], which may account for the differences in radiation responses to subpopulations within the tumor [16]. In addition, tumor hypoxia [17] might be a contributor factor to this phenotype, with radiographically identifiable regions within STS exhibiting poor perfusion and dense cellularity [18–20]. From a prognostic standpoint, tumor hypoxia has been associated with a more aggressive phenotype [21] and a higher risk of metastatic disease [22, 23]. This association may explain the distant control and overall survival benefit observed when FPR is achieved, in addition to the expected locoregional control benefit [24, 25], which may be in part due to the improved R0 (i.e., negative tumor at ink) resection rates in patient that achieve FPR^6 26^. Therefore, efforts to personalize treatment both inter- and intratumorally are required to optimize FPR rates and, in turn, patient outcomes.

Advances in our understanding of tumor heterogeneity, radiosensitivity, and radiomics have provided the opportunity to personalize our radiation treatment planning and provide directed treatment escalation of STS, thereby improving clinical responses and outcomes. These improvements have been facilitated by advances in tumor imaging, much of which is part of the standard workup for STS, including computed tomography (CT) and magnetic resonance imaging (MRI). Less commonly used MRI sequences, such as apparent diffusion coefficient (ADC) and dynamic contrast enhanced (DCE) images, demonstrate cellularity and hypoxia within STS [18–20] and are prognostic for tumor response to radiotherapy [27–32] as early as week two of treatment [28]. By using these sequences, MRI habitat analyses identify cellular subpopulations that may represent a distinct tumor biology [3, 4, 33]. Overlaying of these sequences, which identify cellular density, hypoxia, and perfusion, has allowed for distinction between regions of viable oxygenated, viable hypoxic, and necrotic cells [16, 29, 34, 35]. The focal dose escalations would be limited to regions within the gross tumor volume (GTV), while maintaining the same microscopic dose for the broader clinical target volume (CTV) coverage, without a significant change in dose to the surrounding normal tissue [36]. Therefore, STS is the ideal disease to utilize genomics and radiomics to effectively personalize radiation treatment, optimizing pathologic response, R0 resection, and outcome.

In this study, we describe a prospective clinical trial to test a novel genomic-based RT dose optimization algorithm (GARD), utilizing radiomics-habitat directed targeting, to improve the clinical outcomes for soft tissue sarcoma. GARD-based clinical trial modeling proposes that selectively increasing dose (60 and 70 Gy in 25 fractions) to the radioresistant half of the tumor will triple the number of patients that experience a favorable pathological response, compared to standard of care dose (50 Gy in 25 fractions). To identify which intratumoral regions would benefit from the higher optimized doses, we integrate a radiomic habitat-based approach, directing the GARD-optimized RT dose to the cell dense and hypoxic MRI based subpopulations. This isotoxic approach is hypothesized to result in acceptable normal tissue dosing for the GARD-optimized RT dose, focally escalating radiation dose levels with the use of modern radiation techniques (e.g., simultaneous integrated boost [SIB]). The hypofractionation offers a higher biological effect on these low α/β regions, providing an avenue for safely improving FPR rates without significantly increasing toxicity, which is associated with improved local control, distant control, and overall survival [24–26, 37]. With improved understandings of tumor heterogeneity, radiosensitivity, and radiomics, our goal is to personalize radiation treatment for each patient.

### Aims

The primary aim of the study is to determine whether radiomic habitat directed GARD-optimized RT dose escalation can significantly increase the favorable pathologic response rate by > 3 fold over historic standard neoadjuvant radiotherapy (7.9%) to 24%. In addition to safety and feasibility of this approach, we will investigate predictors of response, radiomics-genomic correlation, circulating biomarkers of response, and the genomic heterogeneity within these habitats to create non-invasive radiomic biomarkers for further prospective validation.

## Methods/design

### Ethics approval

The study is approved by the Institutional Review Board (IRB) of the H. Lee Moffitt Cancer Center and Research Institute (MCC IRB #21,136). The HEAT trial is registered at the US National Institutes of Health (ClinicalTrials.gov) #NCT05301283. The current protocol is version 1.4 dated May 5, 2023.

### Study design

**Fig. 1:**
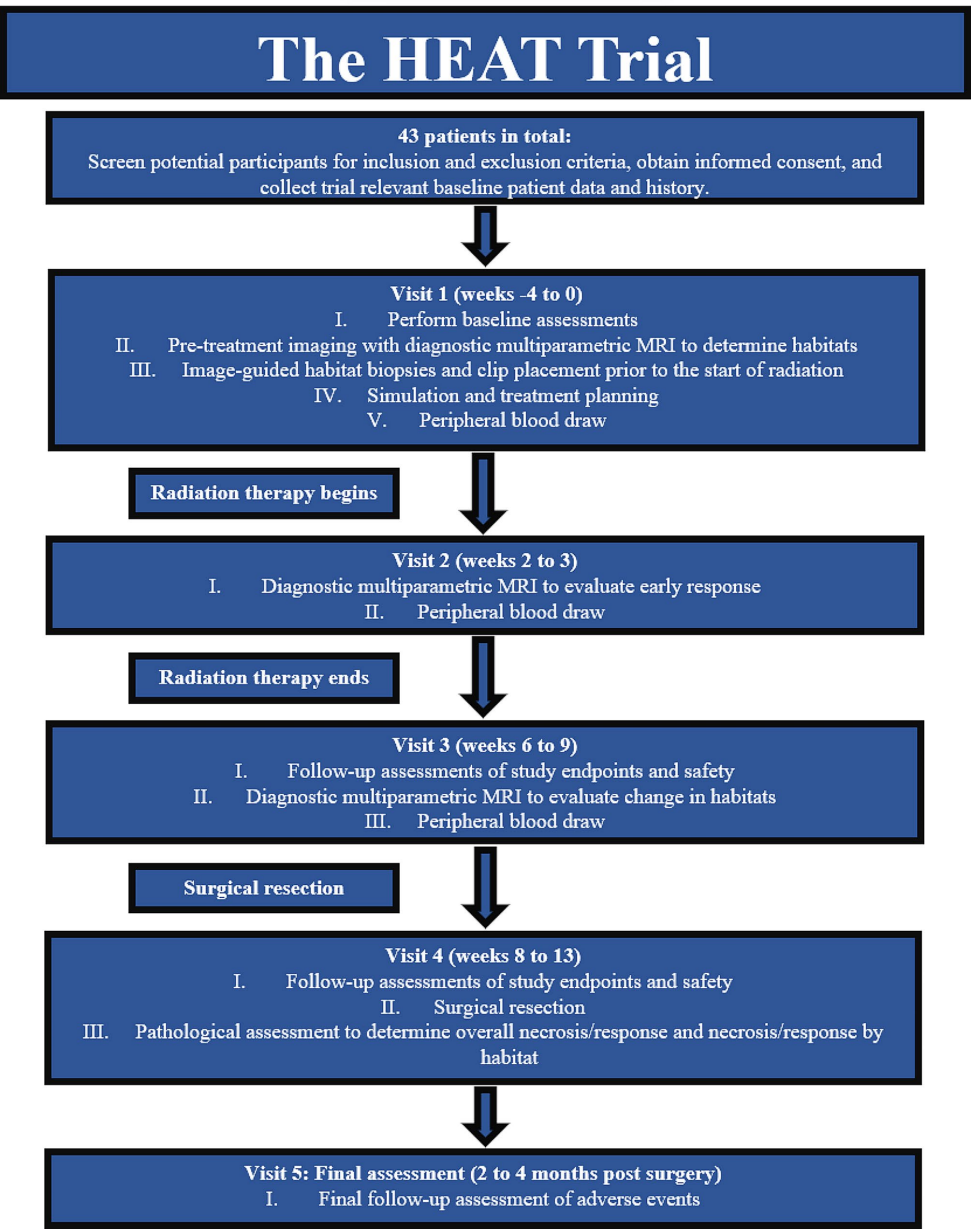
Trial schema. Radiation therapy begins between visit 1 and visit 2. All patients will be seen during weekly treatment visits throughout radiation therapy. Radiation therapy ends between visit 2 and visit 3. Surgical resection occurs between visit 3 and visit 4

This is a non-randomized, single arm, single institution, Phase 2 study of adult patients with non-metastatic high-grade deep STS (grade ≥ 2). The H. Lee Moffitt Cancer Center and Research Institute is responsible for the coordination and trial management, as well as quality assurance including reporting, monitoring, and database management. The study schema is depicted in Fig. 1.

We hypothesize that GARD-optimized RT dose for STS (60 to 70 Gy in 25 fractions), delivered to MRI defined radiomic habitats will triple the FPR rate as defined by response in the surgical specimen of ≥ 95% cellular response. With 36 evaluable patients, this study has 81.8% power to detect an increase in the FPR rate from 7.9% (benchmark selected based on historical data) to 24%, with a one-sided alpha = 0.05. A Z-test with continuity correction will be used to determine changes in the FPR. Assuming 15% of patients will be unevaluable, up to 43 patients will be enrolled within 24 months. This trial uses Fleming’s 3-stage design, in which stage 1 accrual is 13 evaluable patients over 6 months, with a projected accrual rate of 2 patients per month. Stage 2 accrual is 10 and the final stage accrual is 13. Assuming 50% of the eligible patients over a 12-month period enroll in this trial, 3 patients per month will enroll.

### Inclusion and exclusion criteria

Patients with a histologically or cytologically confirmed diagnosis of high-grade (grade ≥ 2) STS of the deep trunk and/or extremity that has been deemed resectable prior to the start of the trial can be included in this study. The main exclusion criteria are metastatic disease, contraindications to MRI, or prior overlapping radiation therapy fields. All inclusion and exclusion criteria are shown in Table 1.

**Table 1:** Inclusion and exclusion criteria

Criteria	Details
Inclusion	1. Provision of signed and dated informed consent form 2. Stated willingness to comply with all study procedures and availability for the duration of the study 3. Age ≥ 18 years 4. For women of childbearing potential: use of highly effective contraception for at least 1 month prior to screening and agreement to use such a method during study participation and for an additional 52 weeks after the end of RT 5. For males of reproductive potential: use of condoms or other methods to ensure effective contraception with partner 52 weeks after RT 6. Agreement to adhere to Lifestyle Considerations (see section 5.3) throughout study duration 7. Pathologically (histologically or cytologically) proven diagnosis of high-grade (grade 2 or 3) STS of the deep trunk and/or extremity. Clinical evidence should be documented, and may consist of pathology or imaging, and should be sufficient to estimate the size of the primary (for T stage) 8. Primary site deemed resectable prior to the start of trial 9. AJCC 8th edition staging T1-4 N0 M0 10. Patients must have clinically or radiographically evident measurable disease at the primary site 11. Pre-RT MRI within 4 weeks of the start of RT 12. ECOG Performance Status 0 to 3 13. Deemed a surgical candidate 14. Patient agrees to blood and plasma preservation for future analysis.
Exclusion	1. Contraindications to an MRI 2. Positive urine pregnancy test 3. Gross total excision of primary STS, including an unplanned excision 4. Superficial sarcoma located primarily in the subcutaneous or cutaneous tissue 5. Evidence of metastatic disease 6. Prior RT to the region of the study cancer that would result in overlap of RT fields 7. Patients with a medical condition or social situation that, at the discretion of the principal investigator, would preclude them from completion of the trial

Abbreviations. AJCC: American Joint Committee on Cancer; ECOG: Eastern Cooperative Oncology Group; MRI: magnetic resonance image; RT: radiation therapy; STS: soft tissue sarcoma

### Objectives

The primary objective of this study is to estimate the FPR of patients with resectable non-metastatic high-grade STS treated with MRI habitat–directed neoadjuvant GARD-optimized RT dose. Secondary objectives are related to the safety and clinical outcomes associated with radiomic habitat-directed radiation dose escalation. Exploratory objectives are concerned with the identification of radiomic biomarkers that correlate with pathologic response that in turn could be used to inform biologic plan adaptation in the future. Biopsy of each habitat will be stored for future genetic testing and correlation with liquid biopsy samples taken at the time of each multiparametric MRI (mpMRI).

#### Primary endpoint

The rate of FPR (tumor response ≥ 95%) at the time of surgery.

#### Secondary endpoints

1. Margin status estimate as defined as the final tumor margin of the surgical specimen will be conducted by pathologist. A clear margin (R0) or a positive margin (R1/R2) will be designated, along with the location of the margin, which will be radiographically correlated to the habitat.
2. Pathologic response of each habitat delineated on pathology specimen as determined by the difference in tumor response by various heterogenous portions of the tumor, as delineated by MRI-directed pre-RT clip placement.
3. Rate and types of adverse events evaluated during weekly on-treatment visits (OTVs) up to 4 months (< 120 days) post-surgery.
4. Disease control, determined as the absence of local tumor progression, to be per assessment of the treating radiation oncologist (i.e., clinical assessment) and radiologist (i.e., radiological assessment) for up to 4 months after surgery.
5. Survival, as assessed by clinical visit, virtual visit, or telephone visit for up to 4 months after surgery.

#### Exploratory endpoints

1. Prediction of tumor response with an early mid-treatment mpMRI at weeks 2 to 3.
2. Collection of tissue habitats for future genomic-radiomic correlations and response to radiation.
3. Collection of liquid biopsies for correlative studies regarding response to radiation.
4. Correlation of diagnostic mpMRI and 0.35 T MRL sequences.

#### GARD modeling of STS

**Fig. 2:**
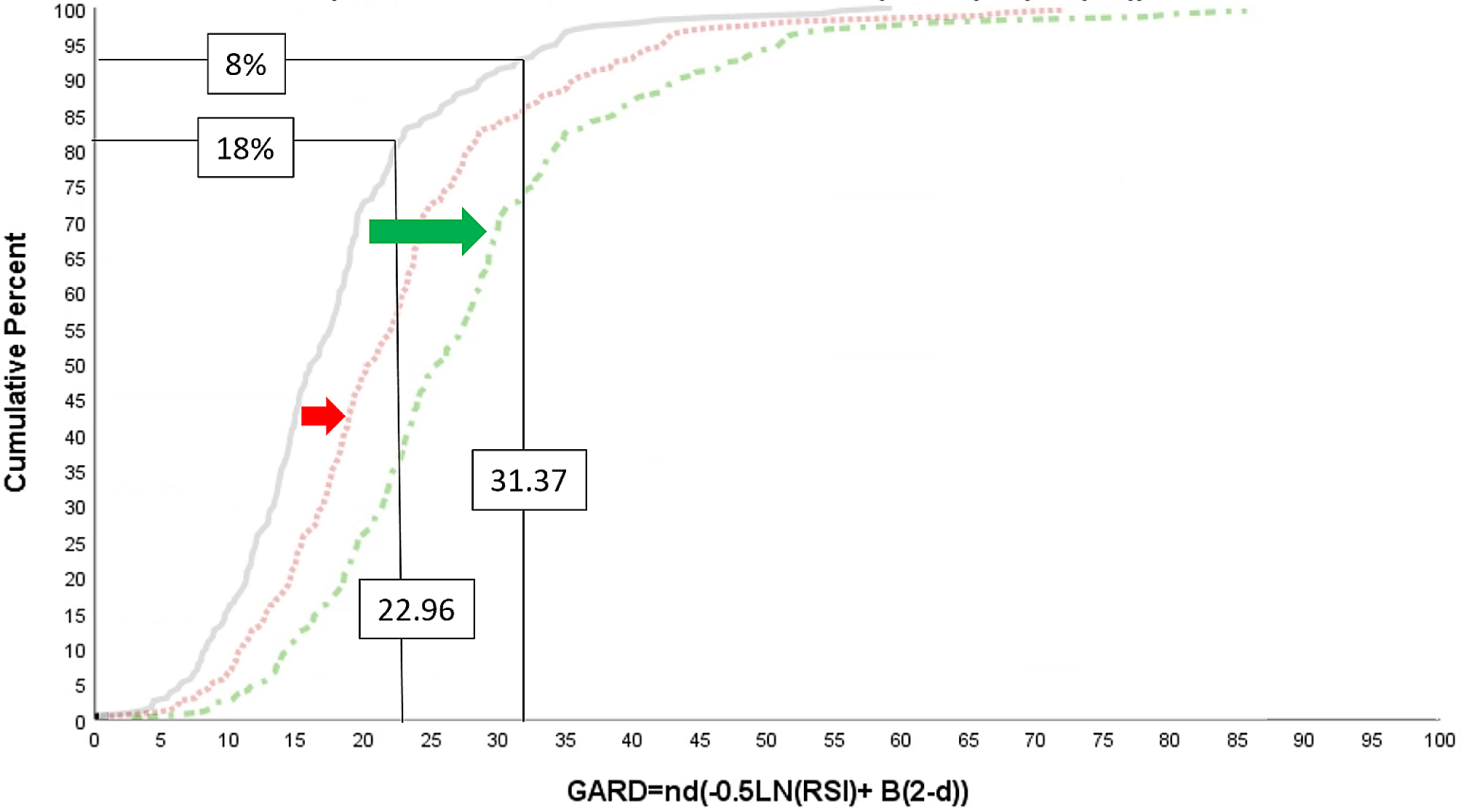
Distribution curve of the cumulative incidence of GARD from 231 STS samples. GARD pathologic response prediction based on historic (8%) and institutional (18%) FPR rates to standard neoadjuvant radiotherapy (50 Gy in 25 fractions), with GARD thresholds of 31.37 and 22.96, respectively. Note the effect that dose escalation with 60 Gy (red) and 70 Gy (green) in 25 fractions has on the percent of patients that are predicted beyond the thresholds. Since both GARD targets are after the linear region in the curve, a uniform dose increase quickly impacts the number of patients that achieve each of the GARD target values. Since habitats are dichotomized based on the gross tumor volumes median value, then 50% of the volume will receive standard dose and the other half will receive dose escalation of either 60 or 70 Gy. Therefore, the probability of achieving a FPR is 8 to 18% for the standard half and an average of 15.2–60.2% to the escalated half of the GTV, would predict for a > 24.3% estimated FPR for the cohort. A tripled FPR rate (8–24%) is a modest estimate that assumes complete dose conformality, with neighboring habitats adjacent to one another, this would be a conservative minimum FPR increase we expect to see clinically. The estimated FPR is assuming the same probability of response for each habitat, but knowing that radioresistant hypoxic regions often require > 30% higher dose for response [40], the radiomic habitat directed approach may better identify the regions that would most benefit from dose escalation, therefore improving overall response

In previous studies we generated the distribution of GARD for 231 STS patients, assuming standard of care neoadjuvant RT dose (50 Gy in 25 fractions), and an average beta of 0.045 [11, 38, 39]. As shown in Fig. 2, GARD ranged from 22.96 to 31.37, with a higher GARD predicting a higher probability of tumor response. In addition, since the GARD calculation is based on the linear-quadratic model, the distribution curve (Fig. 2) has a linear region where there is a rapid increase in GARD. We modeled two possible scenarios. In the first scenario, we assumed that the baseline response to neoadjuvant RT (based on SOC dose) is ∼ 8% (7.9%), as demonstrated in the recent phase 2/3 clinical trial [6]. In a second scenario, a review of our center’s experience revealed that 37 of 202 (18.3%) STS patients treated with standard neoadjuvant RT alone experienced an FPR. Next, we identified approximately the top 8th and 18th percentile values in the GARD distribution (31.37 and 22.96), the highest 8% and 18% radiosensitive sarcomas are most likely to achieve a FPR, which we hypothesize are the GARD target values that optimize clinical outcome. It should be noted that the hypothesized GARD targets are both located after the linear region of GARD suggesting that uniform increase in dose are likely to impact the outcome of these patients. We next modeled the impact on GARD distribution if the dose was 60 and 70 Gy. As shown in Fig. 2, the number of patients that are predicted to achieve each GARD target (31.37 and 22.96) would increase with homogenous dose to 60 Gy (15.2–26.8%) and 70 Gy (26.8–60.2%), depending on true baseline response rate.

#### Radiation simulation and diagnostic mpMRI

**Fig. 3:**
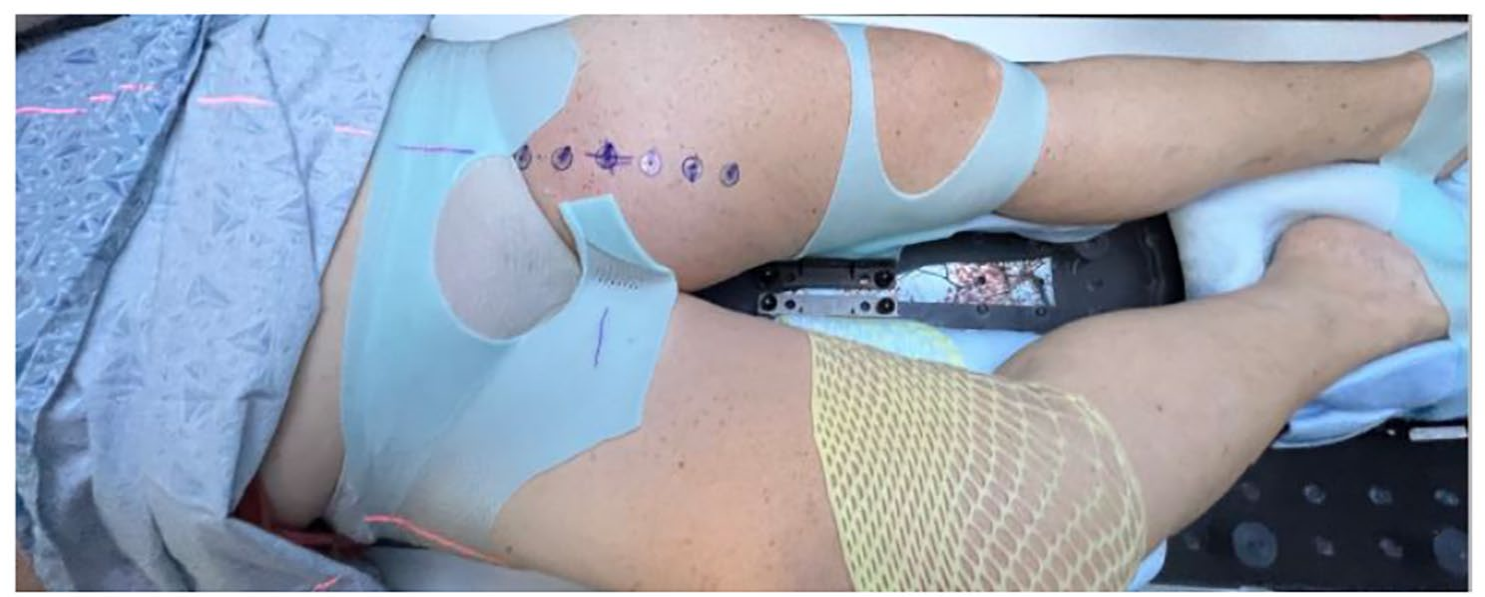
Example of the custom MRI compatible immobilization for a left thigh STS. BB marks on the left anterior thigh will then be tattooed to serve as a guide for setup (e.g., imaging, daily treatment) and habitat directed biopsy that will be used for exploratory genomic studies

A CT simulation will be performed with 3 mm slice thickness that will be used for planning. Simulation will occur with a custom MRI compatible immobilization mold (Fig. 3). The isocenter, along with 1.5 to 3 cm increments superior/inferior along the sagittal plane of the isocenter, will be marked over the extent of gross disease. An additional lateral mark will be placed to control for rotation. The markers utilize a radiopaque BB, which is imaged during the second CT scan, with intravenous (IV) contrast when possible. The BB locations are then tattooed to serve as localization markers during biopsy and scar removal at the time of surgery. Diagnostic mpMRI sequences will all be performed on a Siemens 3T Vida magnet (Siemens AG, Munich, Germany). The mpMRI sequences will be fused to the CT simulation scan and used to identify radiomic habitats for biopsy and direct treatment planning. Patients are treated on combination MRI linear accelerator (MRL) when possible. When treating on the MRL, a true fast imaging with steady-state free precession (TRUFI) [41] sequence will be obtained on the 0.35T MRIdian System (ViewRay Inc., Oakwood Village, OH, USA), that will be used for treatment planning, utilizing the CT from simulation for electron density.

#### MRI radiomic habitat determination, target definitions

**Fig. 4:**
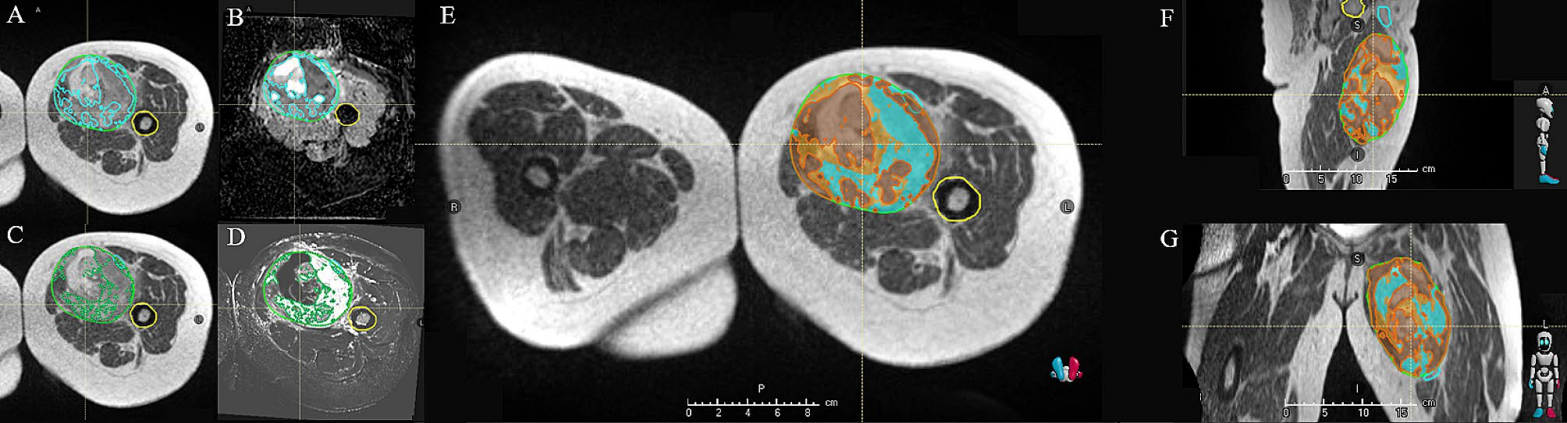
MRL planning shows the GTV on a TRUFI image (**A** and **C**), is rigidly mapped onto an ADC (**B**) and DCE (**D**) sequence to determine the low and high ADC/DCE regions. The regions of high and low overlaps based on median value (**E**: axial view; **F**: sagittal view; **G**: coronal view) are then used to determine habitats 1, 2, and 3

A pretreatment diagnostic mpMRI will be performed to obtain T_2_ short tau inversion recovery (STIR), DWI, and DCE sequences. These sequences will be used to delineate three separate habitats. The gross tumor volume (GTV) will be delineated from the T_1_ post contrast scan, which is combined with STIR (e.g., peritumoral edema), determines the clinical target volume (CTV). This GTV will then be stratified on a per pixel basis to identify three habitats based on ADC intensity (as calculated from DWI) and the rate/magnitude of the DCE (K^trans^). The median value will be used as the dichotomization threshold to define the high (e.g., High ADC) and low (e.g., Low ADC) intensity regions (Fig. 4). The overlay of these sequences will result in three separate and non-overlapping habitats:

1. Habitat 1: Low ADC/Low DCE: high cell density with hypoxia (i.e., the most radioresistant habitat).
2. Habitat 2: Low ADC/High DCE: high cell density that are well oxygenated.
3. Habitat 3:
  - High ADC/Low DCE: necrosis and fluid, low tumor cellularity.
  - High ADC/High DCE: necrosis and fluid, low tumor cellularity.

The three habitats will receive 50 Gy (GTV50), 60 Gy (GTV60), or 70 Gy (GTV70). GTV50 will consist of gross tumor defined by MRI T_1_ post contrast images and the areas of the gross tumor defined above in habitat 3 will receive 50 Gy in 25 fractions. GTV60 will receive the intermediate dose escalation portion of the gross tumor defined by Habitat 2 and GTV70 will receive the high dose escalation portion of the gross tumor defined by habitat 1, with 60 and 70 Gy in 25 fractions, respectively.

The clinical target volumes (CTVs) take neighboring organs at risk (OARs) into account. For habitat 3, the standard radiation dose and fractionation to 50 Gy will encompass the volume of gross and microscopic disease. Typically, the CTV50 will equal the GTV plus a 3 cm margin along the length of the muscle, commonly longitudinal (proximal and distal directions), with a 1.5 cm radial margin expansion, including suspicious edema (as defined on the T_2_ STIR images) up to 4 cm beyond GTV. The CTV margin will respect anatomic barriers (e.g., compartment, bone, skin). CTV60 and CTV70 correlate with habitat 2 GTV60 (i.e., Low ADC/High DCE) and Habitat 1 GTV70 (i.e., Low ADC/Low DCE) volumes, after removing small volumes < 5 cc (or < 0.1 to 1 cm [2]) and subtracting from planning organs at-risk volume (PRV). To minimize toxicity risk, a PRV of normal structures will be created (e.g., bone, joints, organs, major neurovascular structure, and skin surface), expanded either 10 to 15 mm or 5 to 10 mm and contoured out of CTV70 and CTV60, respectively.

An alternative approach can be employed at the discretion of the treating physician, where a single Low ADC volume would be created, combining habitats 1 and 2 for dose escalation. Then small volumes that are < 5 cc (or < 0.1 to 1 cm [2]) would be removed and dosing is based on proximity to OARs. Dosing for the CTV70 would be created from this low ADC volume after excluding the PRV (with a margin of 10 to 15 mm) of normal structures (e.g., bone, joints, organs, major neurovascular structures, and skin surface). CTV60 would then be the remaining volume after removing PRV of normal structures plus 5 to 10 mm.

The PTVs includes the CTV plus error of setup and organ motion. Typically, the PTV will equal the CTV plus 3 to 5 mm depending on setup, but PTV as low as 1 mm can be used if real-time tracking is employed. Daily image-guided radiation therapy (IGRT) is encouraged to minimize daily setup errors, preferably a cone beam CT or MRI. The biopsy clips assist in image-guided localization to ensure accurate daily setup for radiation treatment delivery.

Skin surfaces are not to be contoured in CTVs or PTVs unless grossly involved with tumor, and the use of bolus on the skin is discouraged.

#### Normal tissue constraints

Radiation to normal tissue will be administered under accepted normal tissue tolerances, with efforts made to avoid treating the full circumference of an extremity, anogenitalia (e.g., scrotum, testis, vulva, and anus), skin over commonly traumatized areas (e.g., elbow and knee), and weight-bearing bones (e.g., femoral head and neck) (Table 2).

**Table 2:** Normal structure dose constraints

Structure	Constraint
Anus	V30 Gy < 50%
Genitalia	V30 Gy < 50%
Joint	V50 Gy < 50%
Femur	V50 Gy < 50%
Testes	V3 Gy < 50%
Skin Strip	V20 Gy < 50%
Kidney	V14 Gy < 50%
Femoral head/neck	V60 Gy < 5%
Lung	V20 Gy < 20%
Spinal Cord	D0.03 cc < 45 Gy
Skin minus 5 mm	53.5 Gy (5 cc < 55 Gy)
Other normal structure	Tolerated dose 5/5

Efforts to limit dose near neighboring structures will be made, limiting with no more than 50% of the anogenital structures (e.g., anus and genitalia) receiving 30 Gy; 50% of joints or weight-bearing bones (e.g., shoulder, hip, elbow, femur, and knee) receiving 50 Gy; 50% of a skin strip (longitudinal strip of skin and subcutaneous tissue with a minimum of 2 cm in length) receiving 20 Gy; 50% of the kidney receiving 14 Gy; and 50% of the testes receiving 3 Gy (in patients preserving fertility). No more than 5% of the femoral head/neck should receive 60 Gy. No more than 20% of the lung should receive 20 Gy. For any other normal tissue structures, radiation doses should be limited to the established tolerated dose 5/5 for these structures. In addition to standard structures, additional structures that were monitored included neurovascular structures (e.g., femoral neurovascular bundle, sciatic nerve, lumbosacral plexus), with a maximum point dose (0.03 cc) of 67 Gy. A wound flap, defined as the 3 cm of subcutaneous tissue flanking 3 cm beyond the wire/scar, was limited to a point max of 53.5 Gy and V30 < 50%, when possible. If these constraints cannot be met, then techniques to improve dosimetry will be considered. Variations of these constraints will be acceptable if approved by the treating physician.

#### Treatment planning and plan adaptation

The patients will be planned to undergo neoadjuvant external beam radiation by using the intensity-modulated radiation (IMRT) technique, with a SIB to 70 Gy, 60 Gy, and 50 Gy in 25 fractions for habitats 1, 2, and 3, respectively. Each PTV will be prescribed to cover 95% of the respective volume receiving 100% of the respective prescription dose.

**Fig. 5:**
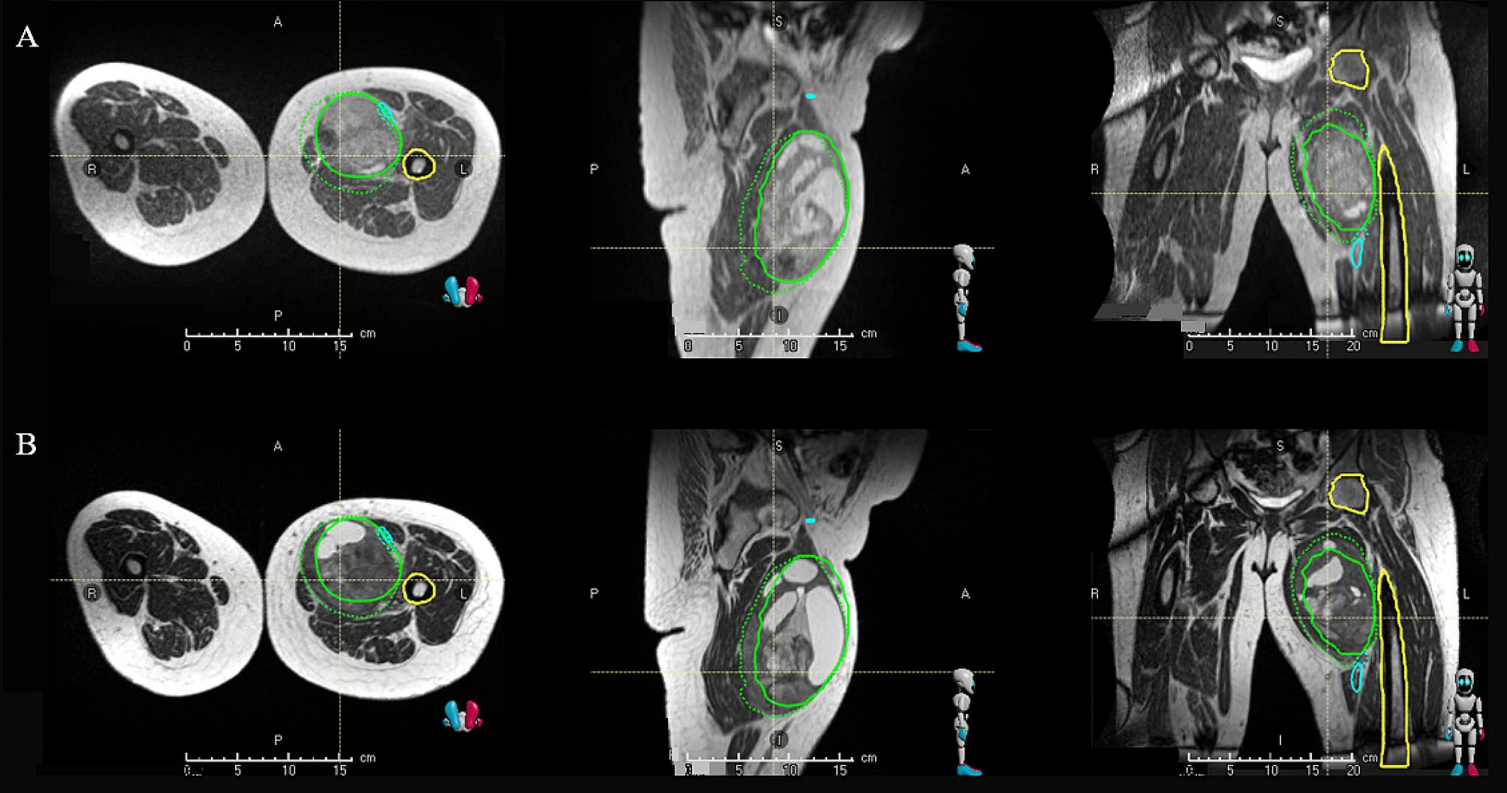
Adaptation at fraction 1 (**B**) to account for tumor growth from the time of simulation (**A**). Solid green line represents GTV at time of simulation and dotted green line represents GTV at time of fraction 1

There is 1 planned adaptations using mpMRI sequences (via Siemens 3T Vida magnet) that occur during treatment, and additional adaptations can be considered for unexpected tumor volume changes, especially day 1 of treatment to account for volumetric changes that occurred from the time of simulation (Fig. 5). The planned adaptation uses a diagnostic mpMRI taken two to three weeks into treatment, where new habitats will be created and dose-adapted based on the tumor’s early response to treatment. To properly account for alterations within the habitats, the initial ADC and K^trans^ median values are then normalized to account for differences between the two scans. The normalization factor for K^trans^ is the average signal for the same adjacent uninvolved vessel (1 cm in length) between the initial and second mpMRI. For ADC, a vial with a known ADC value is placed for each mpMRI, and the average signal for a 1 cm radius sphere is used to normalize between the initial and second mpMRI. The vial contains a 50% concentration of polyvinylpyrrolidone, and the ADC value is calculated to be 278 × 10^− 6^ mm^2^/s assuming an ambient operating temperature of 22 °C (the typical temperature within the vault).

#### MRI habitat-directed biopsy and clip placement

Once the habitats are identified from pretreatment imaging, patients are then referred to interventional radiology for an image-guided biopsy and fiducial placement in the three habitats. This will validate the biopsy locations, aid in daily image guidance during treatment delivery, and assist in pathologic identification of each habitat after surgical resection.

Prior to the day of biopsy, the patient images will be reviewed in tandem by the radiation oncologist and interventional radiologist. On the day of the biopsy, active coordination between the interventional radiology team and the radiation oncology team, including nursing, radiation therapy, and physicians, will ensure real-time targeting of the three habitats from the planned entry sites. The patient’s mold used for CT simulation will be placed on the CT biopsy machine, ensuring the patient is in the exact position as during simulation. The RT planning tattoos, serving as entry sites for the biopsy, will be strategically chosen for each habitat to ensure precise needle insertion and to reduce the risk of seeding. Biopsy will be performed under moderate sedation using intravenous midazolam and fentanyl, as per institutional policy. Scout images will be obtained with radiopaque BBs placed at the same levels as the RT planning tattoos. The biopsy sites are planned to be included within the surgical resection bed to mitigate the risk of seeding, as STS are known to possibly seed biopsy tracts. During the biopsy, each habitat biopsy’s details, including size of needle, number of passes, and type/number of clips, will be meticulously recorded by the radiation oncologist while the interventional radiologist performs the procedure. Each set of clips will represent an intra-tumoral habitat and will be used during daily RT setup to ensure proper target coverage. The biopsy tract images will be used by the surgeon to ensure all needle tracts are resected at the time of surgery. Habitat tissue will be preserved as fresh frozen samples for future studies.

### Radiological and pathological treatment evaluation

Post-RT diagnostic mpMRI will be performed within three to eight weeks after the completion of radiation, with the same immobilization and sequences as the pre-treatment mpMRI. This will be used to assess radiographical treatment response, surgical planning, and for future delta radiomics.

**Fig. 6:**
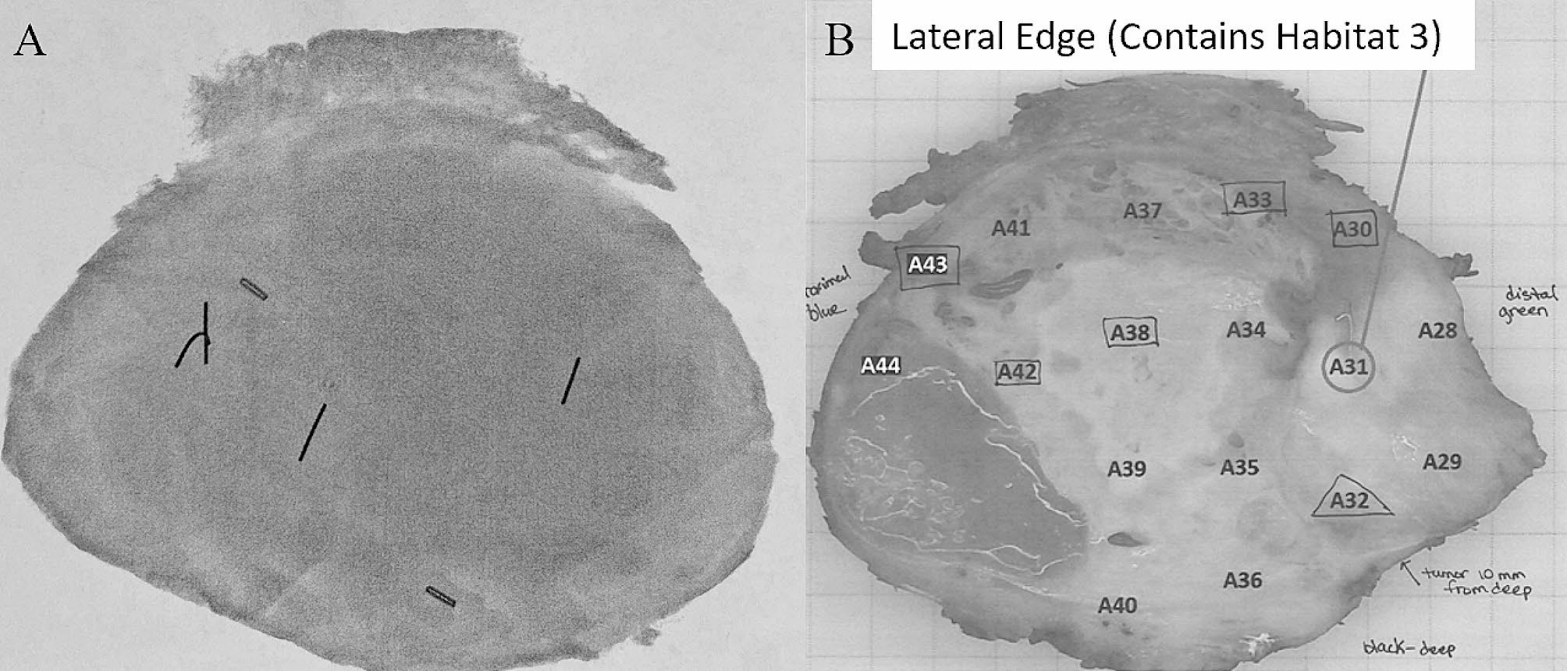
Specimen radiograph displaying markers for each habitat and to help orient with preoperative imaging (**A**). At time of pathological evaluation, the specimen is mapped to a grid to determine percentage of pathological response throughout the specimen (**B**)

After post-treatment mpMRI, resection of the tumor will occur between three to twelve weeks after the completion of radiation therapy. The findings will be correlated to final surgical pathology reports after resection of the tumor and response of each habitat (Fig. 6). The remaining viable cells and margin status will be evaluated by a designated sarcoma pathologist. This evaluation will be used to estimate the pathologic response overall, in addition to the level of response within each habitat. A FPR is 1-% viable malignant cells, with “response” including necrosis, treatment response, and fibrosis.

**Fig. 7:**
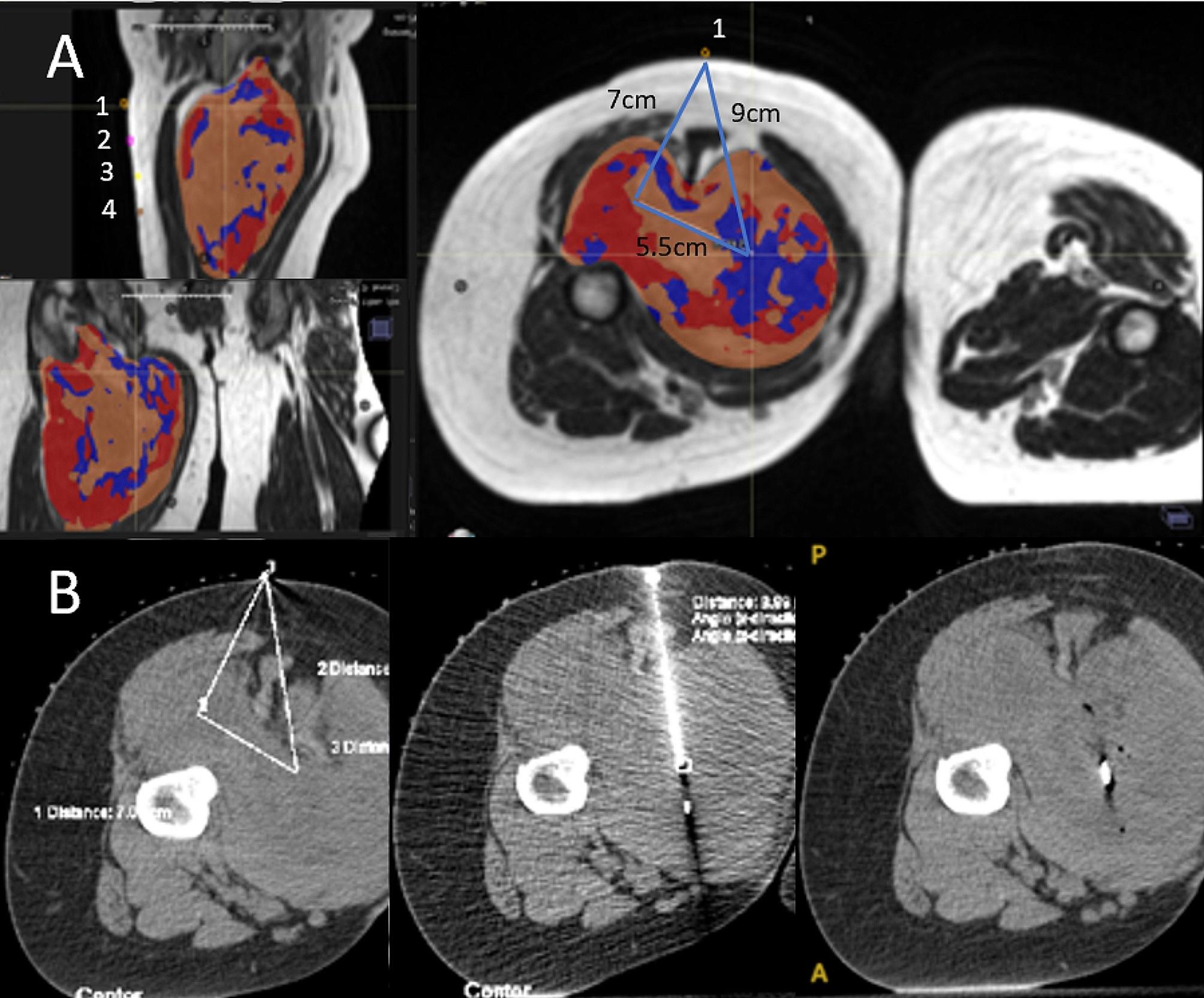
Example of stereotactic biopsy within a habitat of the posterior thigh. (**A**) MRI identification of Habitat 1 (blue) target. To account for sofit tissue rotation, needle insertion is designated at the proximal tattoo (BB#1) and a depth of 7 cm towards the center of the femur is measured, with a medial tangential 5.5 cm line (90 degrees), creating the final biopsy track 9 cm from the surface. (**B**) CT measurements, biopsy, and clip placement are illustrated. Each site is defined by a different marker (e.g., one visicoil proximal biopsy, two visicoils at midpoint habitat, and one visicoil plus one helical mammotome for the distal habitat sampled). In addition to helping with daily set up, these markers will also be used at the time of pathological evaluation to orient the specimen and determine treatment response within each habitat

The pathologists on this study are sarcoma specialized, and cross-review is performed on all cases by a fellow sarcoma specialized pathologist. No molecular markers or assays are required to assess pathologic response, as it is a microscope-based assessment. To help the pathologist ascertain which part of the tumor they are analyzing, the 3 habitats biopsied before the start of RT will be designated with specialized clips in each location (minimum of 1.5-3 cm apart) (Fig. 7). A radiograph is completed on the resected pathologic specimen, to orient the pathologist and determine the habitat locations for intratumoral response analysis. The entire tumor is analyzed for pathologic response along with each habitat marked with clips. The pathologists also mark off the sections that had viable cells remaining (Fig. 6), so future radiomic-dose correlations can be determined, which will account for cumulative dose and spillover.

### Adverse event assessment

Adverse event (AE) and serious AE (SAE) assessment will occur during weekly OTVs, post-RT follow-up (between one to three weeks after completion of RT), and up to four months (< 120 days) after surgery. Grading of AEs will be documented based on Common Terminology Criteria for Adverse Events (CTCAE) v5.0. SAEs will be those that result in death, a life-threatening AE, inpatient hospitalization (> 24 h) or prolongation of existing hospitalization, a persistent or significant incapacity or substantial disruption of the ability to conduct normal life functions. In addition to the assessment of all AEs by CTCAE, surgery AEs are explicitly asked for and collected in detail, including peri-operative and postoperative complications. Major wound complication is defined as a wound that requires more surgery, an invasive procedure (e.g., aspiration), and readmission for wound care and/or IV antibiotic use within 120 days after surgery. In addition, any deep packing (packing to dermis in an area of a dehisced wound measuring 2 cm in length) that is needed for over 120 days will also be included as a major wound complication. Although we are not powered to interpret the long-term sequela, we will continue to follow patients off-trial and beyond 4 months postop, as per NCCN guidelines [42]. The range of two to fourth months postoperative follow up in the trial schema is to account for patients that have evaluation prior to four months and are fully healed, but those patients who have post-operative complications will continue on the trial until four months.

### Statistical considerations

#### Sample size and interim analyses

The primary endpoint is evaluating the FPR rate of patients with resectable non-metastatic high-grade STS treated to GARD-optimized RT dose utilizing MRI habitat–directed neoadjuvant radiation therapy. GARD-based modeling predicted that increasing the dose (60 to 70 Gy) would triple the FPR to an average of > 24.3%. The null hypothesis that the true FPR is 7.9%, which will be tested against a 1-sided alternative hypothesis that the FPR is 24%. We determined the sample size of a maximum of 43 patients using Fleming’s three-stage optimal design, which has a one-sided significance level of 5% and a power of 81.8% if the true FPR is 24% under the alternative hypothesis. The trial is carried out in 3 stages. In the first stage, a total of 13 evaluable patients will be accrued. If there are at least five FPRs among these 13 patients, the study will be considered a success, but will continue to accrue to 23 total patients to obtain further sufficient information about primary and secondary endpoints. Otherwise, it will move onto the second stage that will accrue an additional 10 evaluable patients. If there are two or fewer FPRs in the total of 23 subjects in the second stage, the trial will be stopped for futility. If there are ≥ 5 FPRs among these 23 patients, the study will be considered a success and accrual would end after the 23 total patients. Otherwise, ten additional evaluable patients will be accrued in the final third stage, resulting in a total sample size of 36. The null hypothesis will be rejected if there are ≥ 7 FPRs among these 36 subjects. Considering a dropout rate of 15%, approximately 43 subjects will be enrolled. If the true response rate is 7.9%, then the probability of stopping early for futility is 71.4%, the probability of stopping early for success is 4.4%, and the expected sample size is 26.2. When the trial is considered successful at the first or second interim analysis, a total of 23 evaluable patients will be accrued.

#### Data analysis

The primary outcome, FPR, will be presented as the number and percentage of subjects with favorable response with a 95% confidence interval. For the analysis of secondary endpoints, the frequency and percentage of clear margin and positive margin will be tabulated by the location of the margin. The Fisher’s exact test will be performed to correlate the margin with the habitat. Post-surgery acute wound complications will be tabulated by their types and grades. A swimmer plot will be constructed for the date of onset and the date of the acute toxicity event. Time to event outcomes (e.g., local/regional/distant disease progression/recurrence, disease free survival, and overall survival) will estimated using the Kaplan Meier method.

## Discussion

HEAT is the first clinical trial combining genomics and radiomics to personalize radiotherapy in STS, with the goal of significantly improving treatment response and outcome. This clinical trial utilizes a novel genomic-based algorithm (GARD) and radiomic-habitat directed treatment approach, to safely deliver optimized and escalated isotoxic RT doses (60 or 70 Gy in 25 fractions) to regions of high radioresistance. If successful, this trial would have implications beyond soft tissue sarcoma. We would be demonstrating for the first time that it is possible to improve the clinical outcome of RT-treated patients by integrating biological and radiologic features into clinical trial design and dose determination.

Current studies exploring neoadjuvant treatment intensification for high grade STS are investigating combinational therapies in conjunction with RT. Two such interventional studies that are currently enrolling are the phase 1 “Gemcitabine and Docetaxel With Radiation in Adults With Soft Tissue Sarcoma of the Extremities” study (NCT04037527) and phase 2 “Preoperative IMRT With Concurrent Anlotinib for Localised Extremity or Trunk Sarcoma” study (NCT05167994). An observational study out of Mayo Clinic, “An Imaging Agent (Fluorodopa F 18) With Positron Emission Tomography/​Magnetic Resonance Imaging for Assessing Treatment Response in Patients With High-Grade Soft Tissue Sarcomas” (NCT05560009), is evaluating novel imaging techniques to evaluate RT treatment response. The HEAT trial differs from other contemporary trials because it incorporates genomic and radiomic understanding of STS to craft an individualized and GARD-based treatment to optimize RT dose to radioresistant intratumoral habitats while maintaining clinically acceptable doses to nearby normal structures for high-grade STS.

Understanding STS intra-tumoral heterogeneity and radiosensitivity opens the potential for a more personalized neoadjuvant RT paradigm that utilizes SIBs for GARD-optimized dose adjustments to radioresistant areas of the tumor^18–20,27−32^. Over the past decade, the convergence of radiomics and genomics has given rise to radiogenomics, a discipline that explores the relationship between radiologic phenotypes and genomic intratumor heterogeneity [43–45], which posits that distinct radiological features of tumors are influenced by their underlying biology at the cellular and molecular levels. MRI has demonstrated an ability to identify tumor biology, with radiomic features often predictive of specific genomic markers. Intratumoral heterogeneity can be identified with radiomic habitats [16, 29, 34, 35] that are derived by overlapping different imaging sequences to pinpoint unique radiographic regions in tumors [27–32]. These habitats are then able to serve as biomarkers to predict prognosis and treatment response [24, 25]. The HEAT trial takes advantage of these habitats and uses them to guide GARD-based dose escalation and optimization. Additionally, this approach helps to limit toxicity associated with dose escalation, by only targeting areas known to be the most radioresistant within the actual gross tumor, while allowing the edges of the field that approach or abut critical OARs to receive typical doses as with standard neoadjuvant RT in STS. We hypothesize that this radiomically directed GARD-based dose escalation and optimization will increase the FPR rate by threefold, from a historic 7.9% to an estimated 24.3%.

Prior clinical trials employing uniform dose adjustments (e.g., RTOG 0617, HN005) have not demonstrated improved outcomes for RT-treated patients. In RTOG 0617, uniform RT dose escalation to 74 Gy resulted in decreased OS for NSCLC patients, whereas de-escalation to 60 Gy for HN005 also negatively impacted HPV + oropharyngeal patients [46]. Although clinically unexpected, post-hoc GARD modeling predicted that uniform dose changes applied to all patients would be unsuccessful, whereas pathogenomic-based patient selection for dose escalation/de-escalation would improve patient outcomes. HEAT is the first clinical trial that uses a biological basis for RT dose adjustments, incorporating these predictions into the statistical design of the trial, thus marking a new era for radiation oncology. STS, as identified by GARD, is a disease where these dose optimizations would result in improved treatment response and disease control. The combination of GARD with an MRI radiomic-based habitat identification strategy allows for an intelligent isotoxic SIB approach to these radioresistant intratumoral regions without significant toxicity risk. This is unlike other disease sites (e.g., lung, breast, oropharynx cancer) where only a personalized approach prior to dose adjustments is predicted to impact outcome. GARD predicts that a large treatment benefit would be realized if the dose is selectively increased to 60 or 70 Gy in 25 fractions, a hypothesis being prospectively tested in this trial.

Image sequences commonly obtained for STS workup include CT scans and MRI. Additional functional MRI that may provide insight into tumor biology, include diffusion weighted imaging (DWI), apparent diffusion coefficient (ADC), and dynamic contrast-enhancement (DCE) sequences. DWI is able to evaluate the extent and direction of random water motion in tissue [47], which can aid in determining cellularity, aggressiveness, and response to therapy [30]. An apparent diffusion coefficient (ADC) map can be generated from the DWI by ignoring the contribution of the microcirculation signal. The ADC, or the measured diffusion in tissue, is dependent on the tortuosity of the extracellular space, with lower ADC values associated with increased tumor cellularity and architectural distortion, commonly seen with high tumor grade [48, 49]. A DCE sequence measures rate and magnitude of perfusion and can aid with differentiation between regions of hypoxia, necrosis, and normal perfusion, which correlates to radiographic cellularity and hypoxia in STS [18–20, 50]. By overlaying DCE and ADC sequences, we can identify radiomic habitats based on cellularity and hypoxia, which offer a radiographic representation of the innate tumor biology, allowing us to personalize radiation dose levels within the tumor based on the subclonal region’s phenotype. In addition, tumor hypoxia is more often associated with a more aggressive phenotype [21], a higher risk of metastatic disease [22,23], and resistance to radiation [17].

Furthermore, we also aim to improve understanding of STS radiobiology. For this effort, we will be performing stereotactic biopsies prior to treatment within each habitat and placing a marker. This approach was developed to allow for precise genomic and tissue level evaluation of these different habitat regions and how they respond to different doses. It will allow a comparison between the habitats in terms of their genomics, pathways activated, GARD score, and contribution to circulating tumor DNA (ctDNA). Then each habitat will be assessed to determine individual response to these regions that can be associated with the dose delivered to the given region. Additionally, mpMRIs will also be performed mid treatment during weeks two to three to biologically adapt treatment based upon initial treatment response, and again repeated after the completion of radiotherapy. When treated on the MRL, multiparametric imaging will also be conducted on the MRL to capture both first and second order radiomic features taken before, during and after radiation, as well as daily TRUFI sequences. Along with each mpMRI, pre-treatment, mid-treatment, and post-treatment peripheral liquid biopsy samples will be collected to investigate immunological markers (e.g., peripheral blood mononuclear cells and myeloid derived suppressor cells) and determine if any ctDNA biomarkers are identifiable and how they correlate with treatment response.

Radiomic analysis of the mpMRI data from both the diagnostic and MRL images will be evaluated for their potential to predict tumor response to radiation. Due to the differences in field strength and resolution between diagnostic MRI and MRL images, we will calibrate these images and explore conversion factors, which could enhance the clinical utility and prognostic potential of MRL images. Radiomic data will be analyzed in conjunction with blood, plasma, and tumor genomic data to identify potential radiomic and genomic biomarkers. These biomarkers hold considerable potential to guide future studies to improve personalized radiation therapy for STS, such as improved patient selection, more sophisticated biologically guided RT techniques, and predict patients at high-risk for treatment failure who may benefit from further adjuvant therapies.

Overall, the HEAT trial takes full advantage of current understanding within various fields (i.e., radiomics, genomics, and MRI-guided RT) to push beyond traditional one-size-fits-all RT paradigms. It represents an important step towards biologically and radiographically guided adaptive radiation therapy. If successful, the HEAT trial would provide the first evidence that GARD-based dose adjustments can improve the clinical outcome of patients as predicted, improve our understanding between the relationship between radiomics and genomics, and would open a new era in radiation oncology leading to genomic and radiomic-based clinical trials and practice. In addition, the insights gathered may also enable the identification of novel radiomic and genomic biomarkers that may lead to a refinement of current personalized strategies for STS patients.

## Data Availability

No datasets were generated or analysed during the current study.

## Abbreviations

ADC: Apparent Diffusion Coefficient
AE: Adverse Event
AJCC: American Joint Committee on Cancer
BED_3.29_: Biologically Effective Dose for alpha/beta ratio of 3.29
CT: Computed Tomography
CTCAE: Common Terminology Criteria for Adverse Events
ctDNA: Circulating Tumor DNA
CTV: Clinical Target Volume
DCE: Dynamic Contrast Enhanced
DWI: Diffusion Weighted Imaging
ECOG: Eastern Cooperative Oncology Group
EQD_2_: Equivalent Dose in 2 Gy fractions
FPR: Favorable Pathological Response
GARD: Genomic Adjusted Radiation Dose
GTV: Gross Tumor Volume
HPV: Human Papillomavirus
IGRT: Image-Guided Radiation Therapy
IRB: Institutional Review Board
Ktrans: Volume Transfer Coefficient
MCC: Moffitt Cancer Center
mpMRI: Multiparametric Magnetic Resonance Imaging
MRI: Magnetic Resonance Imaging
MRL: MRI Linear Accelerator
NSCLC: Non-Small Cell Lung Cancer
OARs: Organs At Risk
OTVs: On-Treatment Visits
PRV: Planning Risk Volume
PTV: Planning Target Volume
R0: Negative Tumor at Ink Resection
RSI: Radiosensitivity Index
RT: Radiotherapy
SAE: Serious Adverse Event
SIB: Simultaneous Integrated Boost
STIR: Short Tau Inversion Recovery
STS: Soft Tissue Sarcoma
TRUFI: True Fast Imaging with Steady-State Free Precession
